# Language Dominance in Patients With Malformations of Cortical Development and Epilepsy

**DOI:** 10.3389/fneur.2019.01209

**Published:** 2019-11-21

**Authors:** Giorgi Kuchukhidze, Christian Siedentopf, Iris Unterberger, Florian Koppelstaetter, Martin Kronbichler, Laura Zamarian, Edda Haberlandt, Anja Ischebeck, Margarete Delazer, Stephan Felber, Eugen Trinka

**Affiliations:** ^1^Department of Neurology, Medical University of Innsbruck, Innsbruck, Austria; ^2^Department of Neurology, Christian Doppler Klinik, Paracelsus Medical University of Salzburg, Salzburg, Austria; ^3^Department of Neuroradiology, Medical University of Innsbruck, Innsbruck, Austria; ^4^Department of Radiology, Sanatorium Kettenbrücke, Innsbruck, Austria; ^5^Neuroscience Institute, Christian Doppler Klinik, Paracelsus Medical University of Salzburg, Salzburg, Austria; ^6^Department of Psychology, University of Salzburg, Salzburg, Austria; ^7^Centre for Cognitive Neuroscience, University of Salzburg, Salzburg, Austria; ^8^Department of Pediatrics I, Medical University of Innsbruck, Innsbruck, Austria; ^9^Department of Pediatrics, City Hospital, Dornbirn, Austria; ^10^Institute of Psychology, University of Graz, Graz, Austria; ^11^Department of Diagnostic and Interventional Radiology and Neuroradiology, Gemeinschaftsklinikum Mittelrhein, Koblenz, Germany

**Keywords:** malformations of cortical development, epilepsy, language, functional MRI, epilepsy surgery

## Abstract

**Background:** Language function may be reorganized in patients with malformations of cortical development (MCD). This prospective cohort study aimed in assessing language dominance in a large group of patients with MCD and epilepsy using functional MRI (fMRI).

**Methods:** Sixty-eight patients (40 women) aged 10–73 years (median, 28.0; interquartile range, 19) with MCD and epilepsy underwent 1.5 T MRI and fMRI (word generation task). Single-subject image analysis was performed with statistical parametric mapping (SPM12). Language lateralization indices (LIs) were defined for statistically significantly activated voxels in Broca's and Wernicke's areas using the formula: LI = (*V*_L_ – *V*_R_)/(*V*_L_ + *V*_R_) × 100, where *V*_L_ and *V*_R_ were sets of activated voxels on the left and on the right, respectively. Language laterality was considered typical if LI was between +20 and +100 or atypical if LI was between +19 and −100.

**Results:** fMRI signal was elicited in 55 of 68 (81%) patients. In 18 of 55 (33%) patients, language dominance was typical, and in 37 of 55 (67%) patients, atypical (in 68%, right hemispheric; in 32%, bilateral). Language dominance was not influenced by handedness, electroclinical, and imaging features.

**Conclusions:** In this prospective study on a large group of patients with MCD and epilepsy, about two-thirds had atypical language dominance. These results may contribute to assessing risks of postsurgical language deficits and could assist in planning of “cortical mapping” with intracranial electrodes in patients who undergo presurgical assessment.

## Introduction

Malformations of cortical development (MCD) occur when the normal process of cerebral cortical development including neuronal proliferation, migration, and organization is disrupted (1). The majority of children and adults with MCD have drug-resistant seizures, and epilepsy surgery may render up to 75% of them seizure free (2–6). MCD, however, are often localized in functionally eloquent cortical areas conveying sensory-motor, language, or other higher cognitive functions. Therefore, determining a cortical representation of these functions in the framework of presurgical assessment is necessary to avoid postsurgical deficits. Cortical mapping using intracranial electrodes is still considered the “gold standard” for identifying eloquent cortical areas (7). Functional MRI (fMRI) represents a noninvasive additional tool for lateralizing cortical functions such as language and memory in patients with epilepsy (8). Presurgical fMRI may predict postsurgical language and memory deficits in patients with temporal lobe epilepsy (8). fMRI and electrophysiological studies suggest that cortical functions may be reorganized in patients with MCD. These studies, however, have been performed on relatively small samples of patients with MCD (9–17).

In this prospective cohort study, our goal was to assess language dominance in a large group of patients with MCD and epilepsy using fMRI. A further aim was to perform a correlation analysis between language lateralization and various electroclinical and imaging features.

## Methods

### Participants

Seventy-two patients were recruited at the Departments of Neurology and Pediatrics, Medical University of Innsbruck, Austria.

Only patients with epilepsy and an MRI diagnosis of MCD, who had no seizures for at least 48 h before the fMRI study, were included in the study.

Eventually, 68 (40 women) out of 72 recruited patients comprised the study sample, as in four cases, fMRI data could not be analyzed due to massive motion artifacts exceeding 3 mm for translational and 3° for rotational movements.

The median age of patients at the time of fMRI assessment was 28.0 years [interquartile range (IQR) = 19]. Median verbal intelligence quotient (IQ) was 97.0 (IQR = 22). Patients with a verbal IQ lower than 70 were classified as learning disabled (16 patients, 23%). However, all participants were able to perform the tasks. None of patients had aphasia or dysphasia. All underwent prescan training, and the performance of the task was monitored during scanning, as the patients were instructed to whisper the words, which they had to generate. All patients included in the study were compliant and generated the words during the task.

All patients underwent neurological examination and routine electroencephalogram (EEG) recordings using the 10–20 system. EEG video monitoring (EMU) was performed in 65 of 72 patients (90%). The epilepsy side (left, right or bilateral) was determined based on either EMU data or routine EEG and reported seizure semiology. Epileptiform discharges were assessed on interictal routine EEG.

Seizure types and epilepsy syndromes were diagnosed according to the classification of the International League Against Epilepsy (18, 19). Ten patients underwent epilepsy surgery after fMRI study.

We dichotomized our cohort into two groups with regard to the age of seizure onset: those with the onset of seizures at the age of 6 or earlier were categorized as patients with “early onset;” those with the seizure onset later than the age of 6 were categorized as those with “late onset.”

### Structural MRI

Images were acquired on a 1.5-T MR scanner (Siemens Sonata, Erlangen, Germany) using a Siemens-issued eight-channel head coil. All patients underwent at least two high-resolution MRI using an MRI protocol of our institution for imaging of patients with epilepsy. MRI sequences included T1-weighted spin echo and gradient echo three-dimensional multiplanar reconstruction images with and without intravenous contrast application, axial and coronal T2-weighted turbo spin echo, axial and coronal fluid-attenuated inversion recovery, and diffusion weighted sequences. The thickness of 2 mm was chosen for coronal T2-weighted and fluid-attenuated inversion recovery slices, which were acquired at 90° perpendicular to the long axis of hippocampus. T1-weighted anatomic scans were utilized for each subject as reference in single subject analysis with a spatial resolution of 1 × 1 × 1 mm^3^.

### Functional MRI

fMRI was acquired using T2^*^-weighted sequences of echo planar imaging with the following parameters: repetition time = 4 s, echo time = 60 ms, flip angle (α) = 90°, field of view = 250, 25 slices parallel to intercommisural (AC–PC) plane, matrix size = 64 × 64, thickness = 5 mm, distance factor = 0.25, 98 repetitions, giving a voxel size of 3.91 × 3.91 × 6.25 mm3 covering the whole brain.

### fMRI Task Design

The “word generation” language task was performed: The patients were asked to first generate words belonging to the category “Animals” (active condition 1), then to the category “Tools” (active condition 2), and to rest (resting condition) after each active condition. The task consisted of nine blocks. Every block consisted of active and rest conditions; each condition lasted for 15 s. The instructions were given through earphones.

A series of 98 sequential whole-brain echo planar imaging T2^*^-weighted scans was acquired, consisting of five volumes during active condition (A_1, 2_) alternating with five volumes during rest condition (R) yielding a block order of RA_1_RA_2_RA_1_RA_2_RA_1_RA_2_RA_1_RA_2_RA_1_R. Patients underwent a short training immediately before the study. Subjects were asked to whisper the words to monitor the task performance and decrease motion artifacts that could be induced by speaking loudly. All participants were native German speakers.

### fMRI Data Analysis

Image analysis for revealing significant brain activation based on changes in blood oxygen level dependent (BOLD) signal (20) was performed on each subject's fMRI data using statistical parametric mapping (SPM12, Wellcome Department of Cognitive Neurology, London, UK; http://www.fil.ion.ucl.ac.uk/spm/software/spm12/) under MATLAB 7.4 (MathWorks Inc., Natick, MA, USA; http://www.mathworks.com/). The functional data sets of each patient were motion corrected after discarding the first three volumes to allow signal stabilization. Eventually, 95 volumes per series were utilized for data analysis. Anatomical high-resolution images were coregistered to a mean functional image of each subject. Images were not normalized spatially since the majority of patients had MCD, which distorted brain anatomy. Finally, the functional images were spatially smoothed using an 8-mm full width at half maximum Gaussian kernel. A statistical analysis on the basis of the general linear model was conducted as implemented in SPM12. The delta function of the block onsets was convolved with the canonical form of the hemodynamic response function for a duration corresponding to the block length, to generate the model time courses for the three conditions in each task. A high-pass filter (1/288 Hz) was used to remove low-frequency drifts. No global normalization was used. SPM maps of the contrast of voxels with increased intensity during “active” blocks in relation to the resting state (“rest”) in the whole brain were computed. Clusters of activation were reported as significant when they surpassed an initial threshold of *p* < 0.001 (uncorrected) and had a family-wise error (FWE) corrected *p* < 0.05 on cluster level.

### Language Lateralization

Laterality index (LI) was based on two regions of interest (ROI): (i) a frontal inferior ROI—Broca's area including left inferior frontal gyrus with orbital, triangular, and opercular parts as well as dorsolateral prefrontal cortex (parts of Brodmann's areas 44, 45, 46, and 47), and (ii) a temporo-parietal ROI—Wernicke's area with posterior aspect of superior temporal gyrus, supramarginal, and angular gyri (parts of Brodmann's areas 22, 39, and 40). ROI masks were obtained from Wake Forest University PickAtlas toolbox (21, 22). LIs were defined using the formula: LI = (*V*_L_ – *V*_R_)/(*V*_L_ + *V*_R_) × 100, where *V*_L_ is the set of activated voxels on the left and *V*_R_ is the set of activated voxels on the right. LI values between +19 and −19 were classified as bilateral activation; values between +20 and +100 as lateralization to the left hemisphere and values between −20 and −100 as lateralization to the right hemisphere. ROI LI values were calculated using the LI toolbox for SPM12 with bootstrapping, and weighted mean LI values were utilized for the analysis (23). Patients were divided into two groups with regard to their language laterality profile: (i) those with *typical* language laterality (with left dominant hemisphere): LI between +20 and +100; (ii) those with *atypical* language laterality (with right-hemispheric or bilateral language dominance as determined by registered BOLD signal): LI between +19 and −100.

### Handedness

Handedness was assessed by Edinburgh Handedness Inventory (EHI) (24). EHI quotient was calculated by the following equation: EHI-Q = *R* – *L*/*R* + *L* × 100, where *R* is a score for the right hand and *L* is a score for the left hand. In our institution, patients with the score between +60 and +100 are considered right-handed, those with the score between −60 and −100 are regarded left-handed, and scores between −59 and +59 indicate ambidexterity.

### Verbal IQ

Verbal IQ was assessed by a multiple-choice vocabulary test (25).

The following electroclinical variables were analyzed in relation to the fMRI activation patterns: age at the time of fMRI study, sex, MCD location and laterality, handedness, epilepsy syndrome, laterality of seizure foci, EEG abnormalities, occurrence of learning disability, motor deficit, lifetime history of status epilepticus, age at seizure onset, epilepsy duration, seizure outcome at the time of fMRI, and seizure frequency during the first year of epilepsy.

### MRI Diagnosis of MCD

MCD were diagnosed based on MRI and were classified according to the nomenclature proposed by Barkovich et al. (1). MCD were divided into three categories based on the aforementioned nomenclature: category I—MCD due to abnormal neuronal proliferation [e.g., tuberous sclerosis, focal cortical dysplasia (FCD type II) with balloon cells]; category II—MCD due to abnormal neuronal migration [e.g., periventricular nodular heterotopia (PNH)]; category III—MCDs due to abnormal late migration/cortical organization [e.g., polymicrogyria, FCD without balloon cells (FCD type I)]. Dysembryoplastic neuroepithelial tumor and ganglioglioma were also included in the sample, as they are incorporated in the classification of MCD (1).

### Statistical Analysis

Categorical data were analyzed by means of Fisher's exact probability test (two-tailed) for 2 × 2 tables. Either Freeman–Halton extension of Fisher's exact probability test or chi-square test with Yates correction (if all expected cell frequencies were ≥5) was used for tables larger than 2 × 2. In case of significant differences, paired-wise comparisons were carried out by means of 2 × 2 Fisher's exact probability test or 2 × 2 chi-square test with Yates correction. Noncategorical data (e.g., age at seizure onset) were first analyzed by Kruskal–Wallis test. Two-by-two comparisons were performed by means of Mann–Whitney test. Significance was set at α ≤ 0.05. There were no missing data in the entire analysis.

## Results

Statistically significant fMRI activation (*p* < 0.05, FWE corrected at cluster level) in assessed brain regions (Broca's and Wernicke's areas) were registered in 55 of 68 (81%) patients. Task related statistically significant BOLD signal changes were also registered bilaterally in perirolandic areas as well as in the supplementary motor areas in 60% of cases (this may be attributed to the fact that the patients had to whisper the words). Activated clusters were also found in mesial (55%) and basal (47%) temporal areas, as well as in cerebellum (63%). Clinical and MCD data are detailed in Table 1.

**Table 1:** Electroclinical and imaging features of malformations of cortical development and epilepsy.

**MCD (*****n*** **=** **68)**	
Category 1 (abnormal neuronal proliferation)	24 (35%) FCD II (*n* = 11), TS (*n* = 7), GG (*n* = 3), DNET (*n* = 2), HMGE (*n* = 1)
Category 2 (abnormal neuronal migration)	19 (28%) PNH (*n* = 16), SBH (*n* = 3)
Category 3 (abnormal neuronal organization)	25 (37%) PMG (*n* = 18), FCD I (*n* = 7)
Unilateral	39 (57%)
Bilateral	29 (43%)
Temporal	28 (41%)
Frontal	15 (22%)
Multifocal/along lateral ventricles	14 (21%)
Perisylvian	7 (10%)
Frontoparietal	2 (3%)
Frontotemporal	1 (1.5%)
Insular	1 (1.5%)
**Epilepsy**	
Age at seizure onset, median (interquartile range, IQR) years	12 (15)
Epilepsy duration, median (IQR) years	13 (27)
Medically intractable seizures	47 (69%)
Temporal lobe epilepsy	36 (53%)
Extra- temporal lobe epilepsy	32 (47%)
Febrile seizures	2 (3%)
Status epilepticus	8 (12%)
Diffuse slowing on EEG	26 (38%)
Focal slowing on EEG	53 (78%)
Epileptiform discharges on EEG	35 (51%)
Seizure frequency, 1st year of epilepsy	Daily—8 (12%); weekly—11 (16%); monthly—28 (41%); yearly—21 (31%)

*MCD, malformations of cortical development; FCD II, focal cortical dysplasia type II; TS, tuberous sclerosis; GG, ganglioglioma; DNET, dysembryoplastic neuroepithelial tumor; HMGE, hemimegalencephaly; PNH, periventricular nodular heterotopia; SBH, subcortical band heterotopia; PMG, polymicrogyria; FCD I, focal cortical dysplasia type I; IQR, interquartile range*.

**Figure 1:**
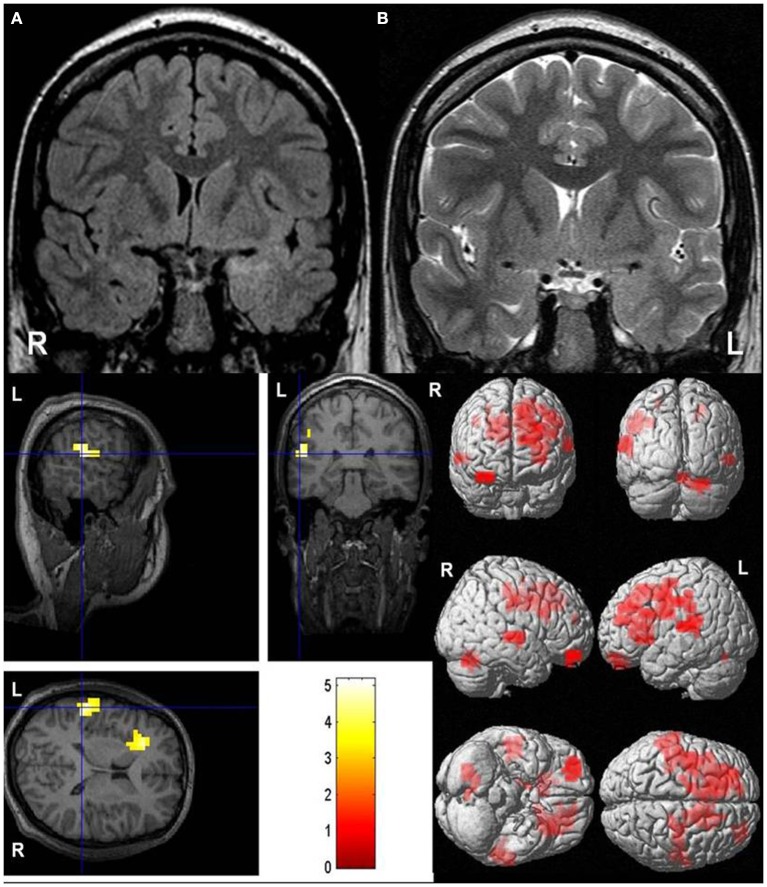
Atypical language dominance in a patient with focal cortical dysplasia type I. **Upper row: (A)** Coronal fluid-attenuated inversion recovery (FLAIR) and **(B)** coronal T2-weighted images show characteristic features of focal cortical dysplasia type I: left (L) temporal lobe is shrunken compared to the right (R) one; there is a higher MR signal in the left temporal lobe (especially in FLAIR sequences) compared to the right one. This patient underwent epilepsy surgery; on histology, focal cortical dysplasia type Ia was diagnosed. **Lower row:** Bilateral language dominance with a lateralization index of +4.9. Blood oxygen level dependent (BOLD) signals are seen bilaterally in Wernicke's areas (threshold *p* < 0.05, family-wise error (FWE) corrected).

**Figure 2:**
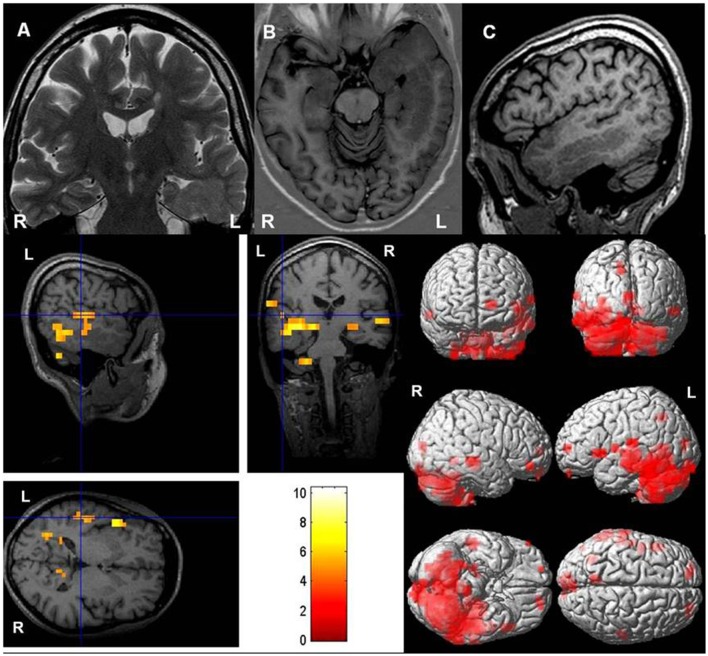
Typical language dominance in a patient with periventricular heterotopia. **Upper row: (A)** Coronal T2-weighted, **(B)** axial T1-weighted inversion recovery, and **(C)** sagittal T1-weighted images show left-sided periventricular heterotopia extending toward cortex. Hippocampus on the left (ipsilateral to periventricular nodular heterotopia) is malrotated. **Lower row:** Left-hemispheric language dominance with a lateralization index of +75. Blood oxygen level dependent (BOLD) responses are mainly in left Wernicke's and Broca's areas [threshold *p* < 0.05, family-wise error (FWE) corrected].

It should be noted that language laterality was determined based only on clusters including Broca's and Wernicke's areas. The median LI was −14 (IQR = 67); it varied over a range from a strong left (+77) to strong right (−72) hemisphere dominance. Using dominance categorical classification, in 18 of 55 (33%) patients, language lateralization was typical (LI between +20 and +100), and 37 of 55 (67%) patients had atypical language lateralization (LI between +19 and −100) (Table S1, Figures 1, 2). Among patients with atypical language dominance, 12 of 37 (32%) patients had bilateral symmetrical language representation and 25 of 37 (68%) had right-hemispheric dominance (Table S1).

In right-handed patients (*n* = 46), the median LI was −15.5 (IQR = 70); 16 of 46 (35%) patients had typical and 30 of 46 (65%) had atypical language dominance. In left-handed patients, the median LI was +1.65 (IQR = 63); two of eight (25%) patients had typical and six of eight (75%) had atypical language representation. The difference between right- and left-handed patients with regard to atypical language dominance was not statistically significant (*p* = 0.460, Fisher's exact test) (Table 2). There was only one ambidextrous patient who had right-hemispheric language dominance.

**Table 2:** Mapping language system in malformations of cortical development (MCD): language lateralization–demographical and clinical data (*n* = 55).

**Demographical and clinical data**	**Typical (*n* = 18)**	**Atypical (*n* = 37)**	**Test**	***p***
Age in years, median (IQR)	31 (12.5)	26 (21)	M-W	0.993
Age at seizure onset in years, median (IQR)	8.5 (12)	15 (19)	M-W	0.398
Age at seizure onset in years, early/late	8/10	10/27	Fischer	0.231
Epilepsy duration in years, median (IQR)	16.5 (20.25)	11 (28.5)	M-W	0.262
Sex, W/M	10/8	23/14	Fischer	0.771
Epilepsy syndrome, TLE/extra-TLE	7/11	13/14	Fischer	0.150
Epilepsy side, R/L/bilat	8/4/6	11/15/11	Fisher	0.370
Seizure frequency during the 1st year of epilepsy, frequent/sporadic	15/3	27/10	Fischer	0.159
Seizure outcome at fMRI time, seizure free/not seizure free	7/11	10/27	Fischer	0.609
Status epilepticus, yes/no	4/14	4/33	Fischer	0.416
Diffuse slowing on EEG, yes/no	10/8	13/24	Fischer	0.244
Focal slowing on EEG, yes/no	13/5	29/8	Fischer	0.738
Epileptiform discharges, yes/no	9/9	22/15	Fischer	0.570
MCD category, 1/2/3	6/6/6	11/10/16	Chi-square	0.775
MCD Location, T/extra-T	5/13	17/20	Fischer	0.249
MCD Side, R/L/bilateral	7/4/7	8/12/17	Chi-square	0.443
Handedness, R/L/ambidexter	16/2/0	30/6/1	Fischer	0.460^*^
Motor deficit, yes/no	6/12	14/23	Fischer	0.775
Learning disability, yes/no	7/11	8/29	Fischer	0.208

*MCD, malformations of cortical development, IQR, interquartile range; W, women; M, men; TLE, temporal lobe epilepsy; T, temporal; R, right; L, left. M-W, Mann–Whitney test; Fischer, Fisher's exact probability test*.

*Fisher's exact probability test (two-tailed) for either 2 × 3 or 2 × 2 tables. Chi-square test for 2 × 3 tables if all expected cell frequencies were ≥5*.

* *Fisher's exact probability test was performed for comparison of R- and L-handers*.

Atypical language lateralization was more common in patients with left- (12/16, 75%) and bilateral (17/24, 71%) MCD compared to those with MCD affecting the right hemisphere (8/15, 53%). The difference, however, did not reach a statistical significance (*p* = 0.443, Fisher's exact test) (Table 2).

A higher rate of atypical language dominance was observed in patients with left-hemispheric (15/19, 79%) or bilateral (11/17, 65%) seizure foci compared to those with right-hemispheric seizure foci (11/19, 58%) without reaching statistical significance (*p* = 0.370, Fisher's exact test) (Table 2).

The median age of seizure onset in the “early onset” group was 2 years (IQR = 2.25 years) and that in the “late onset” group was 17 years (IQR = 12). Atypical language dominance was observed more frequently in the “late onset” group [27/37 (73%)] compared to the “early onset” group [10/18 (56%)]; the difference, however, was not statistically significant (*p* = 0.231).

Results of further statistical analysis (chi-square, Mann–Whitney test) showed that various types and categories of MCD did not differ with regard to language dominance; they also did not differ with this respect if their lobar location was compared (temporal vs. extratemporal). Atypical language lateralization was not determined either by age at the fMRI study or epilepsy duration. Neither seizure frequency during the first year of epilepsy nor seizure frequency at the time of fMRI influenced language dominance. Patients with temporal lobe epilepsy (TLE) and extra-TLE did not differ with respect to the language lateralization. Motor or cognitive deficits did not have significant relationship to the language dominance. These data are detailed in Table 2.

Wada test was performed in nine patients; language dominance determined by Wada test was concordant with the results of fMRI in six of nine patients, with the similarity between fMRI and WADA testing being not statistically significant (*p* = 1.0; McNemar test).

## Discussion

The present study aimed at assessing language dominance in a large group of patients with epilepsy and MCD by means of fMRI. We also focused on correlating language lateralization with electroclinical features. The subjects were recruited from outpatient units of a large public hospital representing a broad spectrum of MCD associated with epilepsy and not a highly selective surgical group. The main finding of the study was a high prevalence (67%) of atypical (bilateral or right hemispheric) language dominance in patients with epilepsy and MCD determined by fMRI. Atypical language lateralization was not influenced by handedness, electroclinical, and imaging features.

In humans, left-hemispheric language dominance is the most common. However, ~6% of the general population has atypical language dominance (26). Different genetic, developmental, environmental, and pathological factors may influence language lateralization (26). Several techniques such as Wada test, fMRI, positron emission tomography, or magnetoencephalography have been used for examining language dominance. In epilepsy patients, there is a much greater variability of language dominance compared to healthy subjects, and it ranges from exclusively left-hemispheric dominance to bilateral symmetric and strong right-hemispheric dominance (26–32). About 30% of patients with localization-related epilepsies exhibit atypical language dominance (30). The factors, which may influence language lateralization in epilepsy patients are left-handedness, familial sinistrality, left seizure focus, and early age at seizure onset (26, 27, 33). The activation patterns in native and acquired languages usually overlap in epilepsy patients; however, the second language has a tendency of being represented in both hemispheres (29). Intra- and interhemispheric language reorganization occurs in patients with epilepsy, especially in left-handed individuals (30%) and those with stroke (30%) (34). In a large fMRI study comparing language dominance in 220 patients with focal epilepsy and 118 healthy volunteers, 24.5% of patients had atypical language activation patterns compared to 2.5% in healthy controls (27). In this group of patients, atypical language dominance was associated with left-handedness, early seizure onset, and vascular pathology on MRI (27). About a third of this population had a normal MRI, 10% had vascular lesions (stroke, cavernomas, arteriovenous malformations), and other lesions included hippocampal sclerosis, tumors, dual pathology, and FCD (27). As opposed to our study, MCD were underrepresented in this paper.

In a more homogenous group of patients with drug resistant TLE (*n* = 162), the highest incidence of atypical (right-sided) language dominance was determined by a combination of left seizure focus with either nonright-handedness (45%) or with early seizure onset (30%) (31). In this fMRI study, patients had either hippocampal sclerosis (36 had left, 30 had right, and 4 had bilateral hippocampal sclerosis), other pathologies (14 had vascular abnormalities, 8 had low grade tumors, and 1 had FCD), or nonlesional MRI (31). In our cohort, there was a slight preponderance of atypical language dominance in left-handed patients and of those with left-sided seizure foci compared to right-handed patients and right-sided seizure foci, respectively. However, these differences were not statistically significant. These associations may be examined in future studies on larger samples. In our cohort, however, the early seizure onset (before the age of 6 years) was not a determinant of an atypical language dominance. In general, the fMRI studies on language dominance in epilepsy include few patients with MCD. The majority of studies are on patients with TLE, and the most common epileptogenic lesions represented in the studies are hippocampal sclerosis, vascular abnormalities, or tumors.

fMRI studies have demonstrated a close association of atypical language dominance with early brain injury (26). The incidence of atypical language representation was as high as 50% when patients had both left-hemispheric early brain injury (before the age of 6 years) and left-sided seizure foci; in those with right-sided seizure foci and right-hemispheric early brain injury, the rate of atypical language lateralization was relatively lower-−37.5% (26). There was a greater incidence of atypical language dominance in left TLE patients (33%) in comparison to right TLE patients or healthy subjects, who had exclusively typical, left-hemispheric language dominance (35). Left hemispheric lesions located near language cortical areas increase the likelihood of atypical language lateralization in children (36). In another study, which investigated the location of receptive language areas by means of magnetoencephalography in epilepsy patients, it was demonstrated that atypical language dominance (or interhemisperic language reorganization) was more common in patients with mesial temporal sclerosis as compared to those with nonmesial–temporal lesions (37). The latter, however, had a higher rate of atypical language lateralization indicated by intrahemispheric reorganization compared to those with mesial sclerosis (37). In summary, atypical language dominance is strongly associated with left-hemispheric lesions and seizure foci as well as early brain injury. This is in line with our findings, as all of our patients who showed atypical language dominance had MCDs (early developmental lesions), high incidence of left-hemispheric and bilateral MCD, as well as left-sided or bilateral seizure foci. We presume that the most likely explanation of a high incidence of atypical language dominance in our population is due to early developmental epileptogenic lesions affecting either left or both hemispheres.

The simple language paradigm used in our study resulted in eliciting of an fMRI signal in 81% (55/68) of patients. A similar observation was made in another study, which mapped different functional modalities (motor, language, visual, memory) in patients with MCD and epilepsy (11): Simple tasks (motor and visual) resulted in fMRI activation in 74% (17/23) and complex tasks (language and memory)—only in 40% (4/10). In the study of Janszky et al. (11), a lower rate of fMRI activations compared to our study could be due to a more severe clinical phenotype of the tested population (all patients had drug-resistant epilepsy, 61% had focal neurological abnormalities, and 52% had mental disability) compared to our patients.

Determining language representation is critical in epilepsy patients who undergo epilepsy surgery. fMRI is a common test for assessing presurgical language dominance in patients with drug-resistant epilepsy. In left TLE patients with left-hemispheric language dominance, the larger was the fMRI activation in the left hemisphere, the greater was the postoperative language deficit after anterior temporal lobe resection (28). These patients underwent early postsurgical reorganization of the language function to the contralateral hemisphere as a compensatory mechanism for regaining language abilities (28). The extent of the resection of the top 10% of the presurgically activated voxels predicted naming decline after temporal lobectomy (32), as it was demonstrated in a study on 35 adult patients with TLE who underwent epilepsy surgery. Right-hemispheric or bilateral dominance were associated with the greater postsurgical language decline (32). In our cohort, till now, only 10 patients underwent epilepsy surgery. The assessment of postsurgical language deficits in this small group and their associations with presurgical fMRI activation patters is not within the scope of this work and is awaiting longitudinal observations. In our study, Wada test was performed only in nine patients who underwent presurgical assessment. Low correlation between the two tests (Wada and fMRI) with regard to language dominance could be due to the mixed population of TLE and extra-TLE patients. It has been shown that patients with extra-TLE may have higher discordance rates between fMRI and Wada test compared to those with TLE (38). In general, congruence of Wada test and fMRI in determining language dominance varies widely from very high—95% (39)—to a relatively low—72.5% in left-sided TLE patients (40). Such variations may be due to sample sizes, patient selection, types of paradigms, lateralization rating, and, eventually, the sensitivity of fMRI for language lateralization.

Language, a cornerstone of human cognition, is a complex, multifaceted mechanism involving dynamic interactions of semantic and syntactic aspects represented in elaborate neural networks (41). In this study, we tested solely an expressive component of the language by utilizing the word generation paradigm, which has also been used by other groups for determining language networks in epilepsy patients (28). Paradigms related to semantic aspects of language, such as picture/auditory naming or semantic decision tasks are also widely used (31, 32, 42–45). Different fMRI tasks, which engage diverse aspects of language, may show either equal or various lateralization patterns (28, 31, 32, 42–45). They may also have different predictive value for postoperative naming decline in epilepsy surgery patients. In a study on 46 patients with temporal lobe epilepsy, preoperative fMRI naming tasks (auditory and picture) were the best predictors of postsurgical naming decline compared to a verbal fluency task (45). Language laterality patterns may also vary in patients with epilepsy and MCD depending on utilized tasks and language components tested. This issue may be addressed in future studies on patients with MCD.

### Limitations

The limitations of this study are related to the fact that MCD diagnosis was mainly based on MRI. There was only a small proportion (10/68, 15%) of patients who underwent epilepsy surgery with subsequent histological diagnosis of MCD. Therefore, we cannot make any inferences about the histological features of the brain tissue in the majority of our patients. Another limitation of the study is the restriction of our cohort to patients with MCD and epilepsy. Subclinical seizure or microseizure activity (46) may contribute to the reorganization of cortical function to an unknown extent. Therefore, we cannot extrapolate our results to patients with MCD without epilepsy.

In this study, we did not analyze out-of-scanner language performance. Therefore, we could not determine the associations between fMRI language dominance and neuropsychological measures of language as has been shown in some studies (47).

## Conclusions and Clinical Implications

In this fMRI study on a large group of patients with MCD and epilepsy, we have demonstrated that the substantial proportion of patients had atypical language dominance. In patients with MCD and drug-resistant seizures who undergo presurgical assessment, the results of the present study may help in assessing risks of postsurgical language deficits and could assist in planning “cortical mapping” with intracranial electrodes.

## Data Availability Statement

All datasets generated for this study are included in the article/Supplementary Material.

## Ethics Statement

The studies involving human participants were reviewed and approved by Ethics Committee of the Medical University of Innsbruck, 35, Anichstrasse, 6020 Innsbruck, Austria. Written informed consent to participate in this study was provided by the participants' legal guardian/next of kin.

## Author Contributions

GK and CS contributed significantly to conception and design of the presented paper, acquisition, analysis and interpretation of the data as well as drafting of the paper. LZ, IU, FK, and EH contributed to acquisition and analysis of data and revising the paper for intellectual content. AI, MD, SF, and ET contributed significantly to conception of the study, interpretation of the results, and gave final approval of the submitted version of the manuscript. MK contributed significantly to the analysis of fMRI data, interpretation of the results, revising the paper, and final approval of the revised manuscript.

### Conflict of Interest

The authors declare that the research was conducted in the absence of any commercial or financial relationships that could be construed as a potential conflict of interest.
