# Supplementary material for: Language Dominance in Patients With Malformations of Cortical Development and Epilepsy
Source: Front Neurol. 2019 Nov 21;10:1209. doi: 10.3389/fneur.2019.01209 (PMC6881376; doi:10.3389/fneur.2019.01209)
Supplement: Supplementary file 1 [file Table_1.DOCX]

**Table e-1. Language dominance in malformations of cortical development**

| **ID** | **age** | **sex** | **MCD** | **MCD Category** | **Handedness** | **Language Activation Side** | **LI** | **Language Dominance** |
| --- | --- | --- | --- | --- | --- | --- | --- | --- |
| P1 | 55 | M | DNET | 1 | Right | no activation | n/a | n/a |
| P2 | 16 | M | DNET | 1 | Left | no activation | n/a | n/a |
| P3 | 25 | W | FCD II | 1 | ambidexter | no activation | n/a | n/a |
| P4 | 22 | M | FCD II | 1 | Right | Left | +68 | Typical |
| P5 | 10 | M | FCD II | 1 | Left | Bilateral | 2.0 | Atypical |
| P6 | 43 | W | FCD II | 1 | Right | no activation | n/a | n/a |
| P7 | 40 | W | FCD II | 1 | Right | Right | -42 | Atypical |
| P8 | 18 | W | FCD II | 1 | Right | Left | +62 | Typical |
| P9 | 24 | W | FCD II | 1 | Right | no activation | n/a | n/a |
| P10 | 30 | W | FCD II | 1 | Right | Bilateral | -17 | Atypical |
| P11 | 16 | W | FCD II | 1 | Left | Right | -32 | Atypical |
| P12 | 19 | W | FCD II | 1 | Right | Left | +55 | Typical |
| P13 | 36 | W | FCD II | 1 | Left | Left | +42 | Typical |
| P14 | 41 | W | GG | 1 | Right | Left | +54 | Typical |
| P15 | 26 | W | GG | 1 | Left | no activation | n/a | n/a |
| P16 | 37 | M | GG | 1 | Right | Right | -24 | Atypical |
| P17 | 20 | W | HMGE | 1 | Right | Bilateral | -19 | Atypical |
| P18 | 30 | M | TS | 1 | Right | Left | +29 | Typical |
| P19 | 23 | W | TS | 1 | Right | no activation | n/a | n/a |
| P20 | 20 | W | TS | 1 | Right | Right | -34 | Atypical |
| P21 | 21 | M | TS | 1 | Right | Right | -28 | Atypical |
| P22 | 20 | W | TS | 1 | ambidexter | Right | -72 | Atypical |
| P23 | 31 | M | TS | 1 | Right | Right | -54 | Atypical |
| P24 | 19 | M | TS | 1 | Right | Right | -41 | Atypical |
| P25 | 22 | M | PNH | 2 | Left | Right | -29 | Atypical |
| P26 | 51 | M | PNH | 2 | Right | Right | -22 | Atypical |
| P27 | 22 | M | PNH | 2 | Right | no activation | n/a | n/a |
| P28 | 26 | W | PNH | 2 | Right | Bilateral | -2.7 | Atypical |
| P29 | 42 | W | PNH | 2 | Right | Right | -44 | Atypical |
| P30 | 27 | M | PNH | 2 | Right | no activation | n/a | n/a |
| P31 | 55 | M | PNH | 2 | Right | no activation | n/a | n/a |
| P32 | 55 | W | PNH | 2 | Right | Right | -37 | Atypical |
| P33 | 37 | W | PNH | 2 | Right | Right | -37 | Atypical |
| P34 | 17 | M | PNH | 2 | Right | Left | +64 | Typical |
| P35 | 29 | M | PNH | 2 | Right | Left | +38 | Typical |
| P36 | 36 | M | PNH | 2 | Right | Right | -44 | Atypical |
| P37 | 26 | M | PNH | 2 | Right | Right | -31 | Atypical |
| P38 | 55 | W | PNH | 2 | Left | Right | -45 | Atypical |
| P39 | 45 | W | PNH | 2 | Right | Left | +75 | Typical |
| P40 | 22 | W | PNH | 2 | Right | Right | -31 | Atypical |
| P41 | 41 | W | SBH | 2 | Right | Left | +24 | Typical |
| P42 | 22 | W | SBH | 2 | Right | Left | +26 | Typical |
| P43 | 32 | W | SBH | 2 | Right | Left | +21 | Typical |
| P44 | 52 | M | FCD I | 3 | Right | Right | -38 | Atypical |
| P45 | 16 | W | FCD I | 3 | Right | Right | -43 | Atypical |
| P46 | 22 | W | FCD I | 3 | Right | Right | -54 | Atypical |
| P47 | 35 | W | FCD I | 3 | Right | Right | -31 | Atypical |
| P48 | 32 | W | FCD I | 3 | Right | Right | -31 | Atypical |
| P49 | 26 | W | FCD I | 3 | Right | Bilateral | +17 | Atypical |
| P50 | 19 | M | FCD I | 3 | Right | Bilateral | +4.9 | Atypical |
| P51 | 12 | W | PMG | 3 | Right | Bilateral | +13 | Atypical |
| P52 | 15 | W | PMG | 3 | Left | Bilateral | +1.3 | Atypical |
| P53 | 42 | M | PMG | 3 | Right | Bilateral | +6.8 | Atypical |
| P54 | 26 | M | PMG | 3 | Right | Right | -54 | Atypical |
| P55 | 32 | M | PMG | 3 | Right | Left | +77 | Typical |
| P56 | 27 | W | PMG | 3 | Right | Left | +61 | Typical |
| P57 | 42 | M | PMG | 3 | Right | Right | -52 | Atypical |
| P58 | 20 | M | PMG | 3 | ambidexter | no activation | n/a | n/a |
| P59 | 57 | W | PMG | 3 | Right | no activation | n/a | n/a |
| P60 | 36 | W | PMG | 3 | Left | Bilateral | +2.3 | Atypical |
| P61 | 61 | W | PMG | 3 | Right | Right | -44 | Atypical |
| P62 | 73 | W | PMG | 3 | Right | Right | -45 | Atypical |
| P63 | 33 | M | PMG | 3 | Right | Left | +52 | Typical |
| P64 | 52 | W | PMG | 3 | Right | no activation | n/a | n/a |
| P65 | 24 | W | PMG | 3 | Left | Left | +42 | Typical |
| P66 | 32 | M | PMG | 3 | Right | Left | +26 | Typical |
| P67 | 34 | M | PMG | 3 | Right | Left | +44 | Typical |
| P68 | 24 | W | PMG | 3 | Right | Bilateral | -14 | Atypical |

Abbreviations: ID– identification number of a patient; MCD– malformations of cortical development; BOLD– blood oxygen level-dependent; LI– lateralization index; n/a– not applicable; DNET– dysembrioplastic neuroepithelial tumour; FCD II– focal cortical dysplasia type II; GG– ganglioglioma; HMGE– hemimegalencephaly; TS– tuberous sclerosis; PNH- periventricular nodular heterotopia; SBH– subcortical band heterotopia; FCD I – focal cortical dysplasia type I; PMG – polymicrogyria; W– women; M– men.
